# Noise, multisensory integration, and previous response in perceptual disambiguation

**DOI:** 10.1371/journal.pcbi.1005546

**Published:** 2017-07-10

**Authors:** Cesare V. Parise, Marc O. Ernst

**Affiliations:** 1 Oculus Research, Redmond, Washington, United States of America; 2 Cognitive Neuroscience Department and Cognitive Interaction Technology-Center of Excellence, Bielefeld University, Bielefeld, Germany; 3 Applied Cognitive Psychology, Ulm University, Ulm, Germany; Technische Universitat Chemnitz, GERMANY

## Abstract

Sensory information about the state of the world is generally ambiguous. Understanding how the nervous system resolves such ambiguities to infer the actual state of the world is a central quest for sensory neuroscience. However, the computational principles of perceptual disambiguation are still poorly understood: What drives perceptual decision-making between multiple equally valid solutions? Here we investigate how humans gather and combine sensory information–within and across modalities–to disambiguate motion perception in an ambiguous audiovisual display, where two moving stimuli could appear as either streaming through, or bouncing off each other. By combining psychophysical classification tasks with reverse correlation analyses, we identified the particular spatiotemporal stimulus patterns that elicit a stream or a bounce percept, respectively. From that, we developed and tested a computational model for uni- and multi-sensory perceptual disambiguation that tightly replicates human performance. Specifically, disambiguation relies on knowledge of prototypical bouncing events that contain characteristic patterns of motion energy in the dynamic visual display. Next, the visual information is linearly integrated with auditory cues and prior knowledge about the history of recent perceptual interpretations. What is more, we demonstrate that perceptual decision-making with ambiguous displays is systematically driven by noise, whose random patterns not only promote alternation, but also provide signal-like information that biases perception in highly predictable fashion.

## Author summary

Sensory information is generally ambiguous, and a single sensory modality most often cannot provide enough information to univocally specify the actual state of the world. A primary task for the brain is therefore to resolve perceptual ambiguity. Here we use a dynamic audiovisual ambiguous display embedded in noise to investigate the computational mechanisms of perceptual disambiguation. Results demonstrate that the brain first extracts visual information for perceptual disambiguation through motion detectors. Such information is next combined with auditory information–and memory of recent perceptual history–through weighted averaging to determine the final percept. This study revealed the particular spatiotemporal stimulus patterns that elicit a stream or a bounce percept, respectively, and it demonstrates that perceptual disambiguation is majorly affected by noise, whose random spatiotemporal patterns provide signal-like information that bias perception in a very systematic fashion.

## Introduction

Perception is well described as an inference process based on noisy and often ambiguous sensory signals. As such, a single sensory modality most often cannot provide enough information to univocally specify the actual state of the world. Throughout the history of vision science, numerous ambiguous displays have been put forward where the very same sensory stimulus allows for multiple, and clearly distinguishable, alternative interpretations—multistable stimuli such as the Necker Cube, the stream-bounce display and binocular rivalry [1–4]. However, in our daily lives perception seems to be surprisingly devoid of such ambiguities. This is because in most real-life scenarios, the brain can rely on a large variety of information that is often not present in the minimalistic ambiguous displays used in laboratory settings. Information for perceptual disambiguation can come from different cues derived from the same or other senses [5], or it may come in form of prior knowledge representing the statistical regularities found in the natural world [6, 7], or recent perceptual history [8–15].

A notable example of ambiguous stimulus is the stream-bounce display (cf. S1 Movie): two identical objects moving towards each other along the same trajectory can be perceived as streaming through each other, or as colliding and bouncing away from one another [1]. Vision alone does not provide enough information to rule out any possible interpretation, and over repeated presentations the two percepts alternate in a seemingly arbitrary fashion. However, if a sound is presented around the time of the crossing, humans are more likely to perceive a bounce [5]. This finding demonstrates that humans integrate multisensory information for perceptual disambiguation, that is, to infer the most likely interpretation of the sensory data. However, the underlying mechanism of this inference process is still poorly understood. What drives perceptual decision making, and which strategy does the brain use to extract and combine information from the different senses?

Sensory information is corrupted by noise arising in the brain at any stage of processing [16, 17]. Therefore, a possible reason for perceptual alternation (in cases where the two states are about equally likely) relies on the random fluctuations of the internal noise [18]. The role of noise on ambiguous displays has been widely investigated over the years; for example, motion coherence (i.e., noise in the motion signal) can reliably predict the time-course of perceptual switches in binocular rivalry with moving stimuli [19]. Likewise, internal noise is at the heart of most computational models for perceptual alternation in binocular rivalry [19–24]. Specifically, perceptual alternation is often interpreted in terms of a double-well energy landscape [24], where both adaptation-recovery and noise contribute perceptual shifts [21]. Moreover, the effects of noise on binocular rivalry have been successfully simulated with biologically plausible spiking neuron models [20, 23].

While these models can predict perception in binocular rivalry given the statistical properties of the noise, it is currently unclear how the individual instances of the noise affect perceptual disambiguation. For example, the spatiotemporal patterns present in the noise may contain signal-like information that systematically biases perception towards one specific interpretation (and against its alternative). Unfortunately, scientists do not have direct access to the noise that is present within the brain, thus making it hard to test this hypothesis. To overcome this problem, and systematically study the effects of noise on perception, psychophysicists often try to override the noise in the system by introducing (external) noise directly in the stimulus [25]. Given that noise does not come with a label, the brain often cannot infer its internal vs. external nature, so it is reasonable to assume that the brain usually treats these two sources of noise in the same way [17], (though see [26] for a recent finding on how the brain may sometimes be capable of distinguishing, and filtering out, different types of noise). Once the exact properties of the noise are known, reverse correlation techniques [27–29] can be used to investigate whether the brain looks for certain patterns in the noise that might help resolving ambiguity. That is, by investigating how the distribution of external noise on each given trial biases perception, it becomes possible–by averaging the classified stimuli and their noises–to estimate the spatiotemporal decision template used for perceptual disambiguation. In turn, this allows one to explore a number of important aspects of perceptual disambiguation, such as what biases perception, and how sensory information is combined across the senses–and over time and space–to determine the final percept.

In the present study we used reverse correlation techniques to investigate the multisensory mechanisms for perceptual disambiguation in the stream-bounce display. Research on multisensory integration has demonstrated that when integrating redundant and unambiguous information from different sensory modalities, the brain operates in a statistically optimal fashion by taking a weighted average of the individual sensory cues. Thereby, the weights are assigned according to the precision of each cue [30]. This solution is statistically optimal because it provides the most accurate and precise perceptual estimate given the noisy sensory information as input. In the case of the stream-bounce display, however, the information provided by vision and audition is complementary in nature. More specifically, while vision informs us about the spatiotemporal trajectories of the moving objects (while information about the nature of the impact is ambiguous: present or not), audition informs us about the presence of an impact by an appropriately timed sound (or the absence of an impact if the sound is absent or presented with inappropriate timing). That is, vision and audition provide information in qualitatively different formats, which cannot be directly averaged without further transformations (e.g., see [31]). The aim of the present study is to characterize how the brain extracts, transforms, and combines complementary information within and across the senses to resolve perceptual ambiguity.

## Results

### Experiment 1

**Fig 1:**
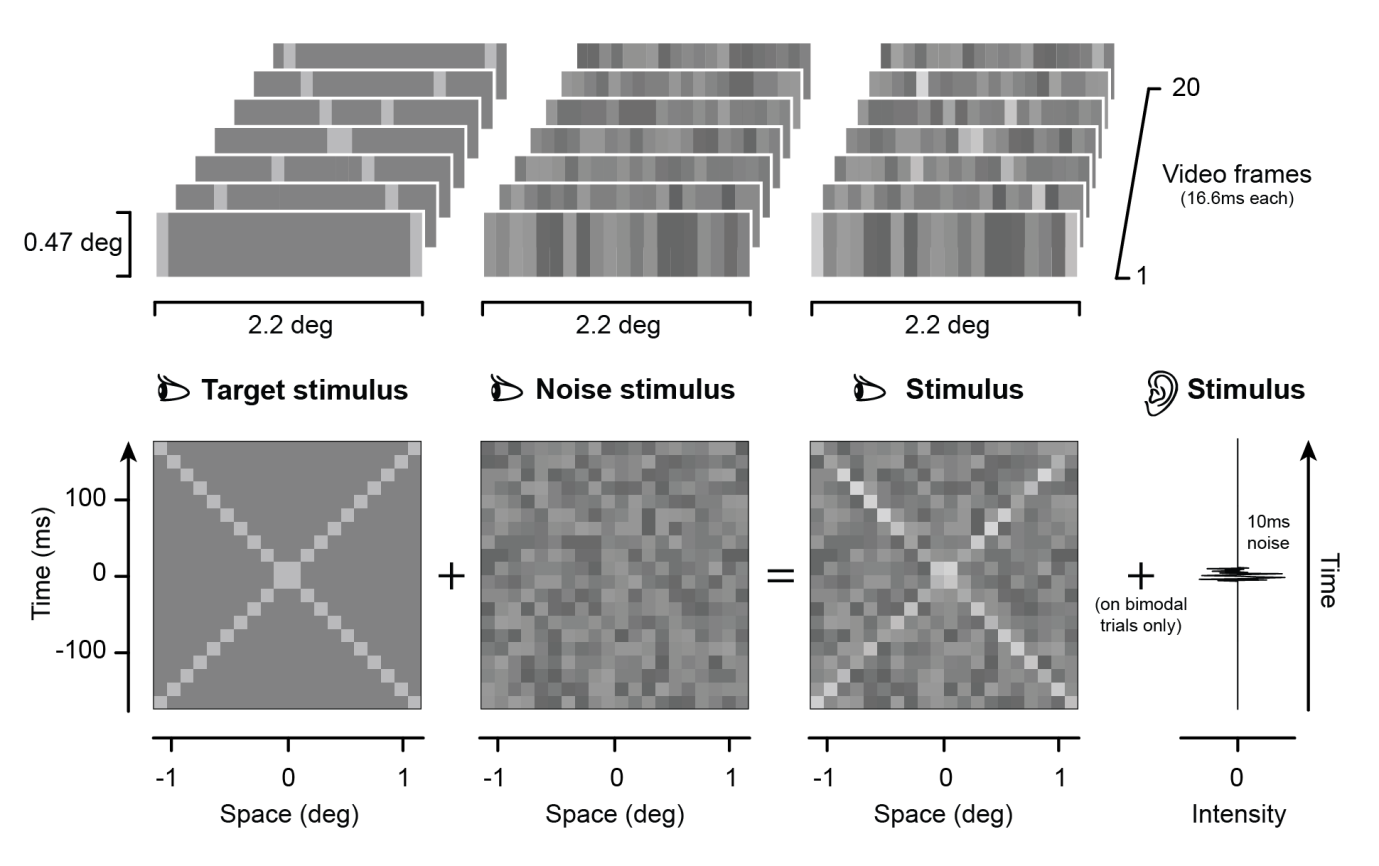
Stimuli. The visual stimuli consisted of two light gray bars moving in opposite directions at constant speed (see also S2 Movie). The moving bars were embedded in dynamic visual noise, and on half of the blocks, a white noise click was presented at the time of the crossing of the moving bars.

Three participants (the author CP, age 33, male; and two female naïve observers, CG and VL age 24 and 23, respectively) were presented with two small vertical light gray bars (0.085° x 0.426° each) moving horizontally along the same trajectory in opposite directions, crossing at the center (signal: Fig 1, left). The moving stimuli were embedded in dynamic visual noise (noise: Fig 1, center; signal+noise: Fig 1, right; S2 Movie) randomly increasing or decreasing in luminance from the middle grey background. In a forced choice task, participant had to report whether they perceived the bars as streaming across each other or as colliding and bouncing off each other (see Methods). The experiment consisted of ~10,000 trials per participant with the dynamic visual noise randomly varying across trials. In half of the trials, a sound (10ms white noise click) was presented at the time of the crossing. Sound and no-sound trials alternated throughout the experiment in blocks of 40 trials.

**Fig 2:**
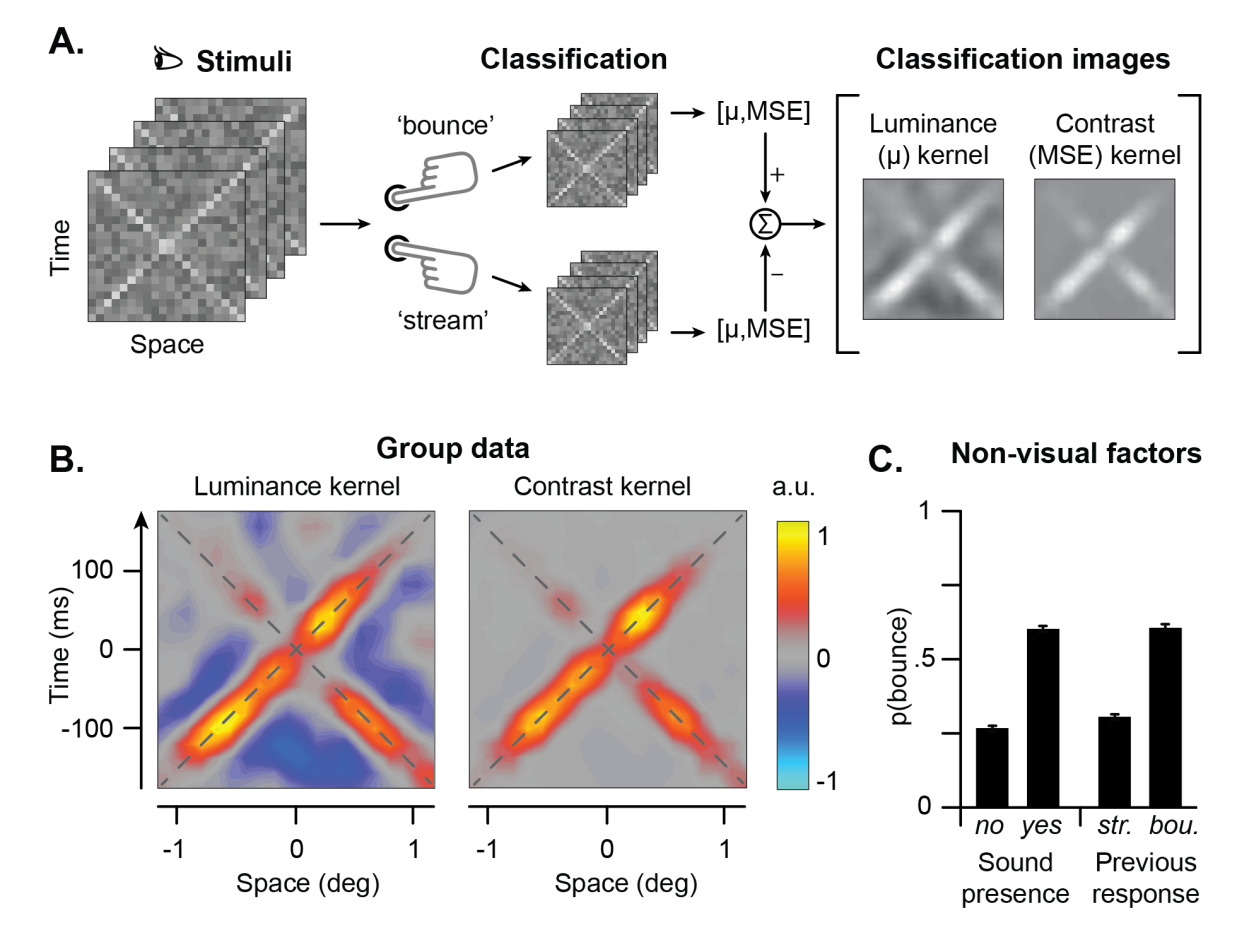
Analyses and results. **A**. Reverse correlation analyses. Noisy stimuli were classified according to participants’ responses, and classification images were calculated from the mean (i.e., luminance) and the mean squared error (i.e., contrast) of each stimulus plus noise sample (see Methods). **B**. Luminance and contrast kernels for the aggregate observer. Warm colors represent samples positively associated to bounce responses, whereas cold colors represent samples negatively associated to a bounce response. **C**. Non-visual factors influencing participants’ responses for the aggregate observer. Errorbars represent the 99% confidence interval. See S1 Fig for individual observers’ data.

Overall, participants perceived the stimulus as bouncing on 43% of the trial (CP: 48%; CG: 40%; VL: 42%). In line with previous studies, sounds significantly modulated participants’ responses and systematically biased the percept toward a bounce: only 27% of the trials without sound were perceived as bouncing, against 61% of the trials with sounds (Fig 2C). Also, participants had a strong tendency towards interpreting the stimulus just as they did in the previous trial (Fig 2C). Such a perceptual stability over time is a classic finding in the study of ambiguous displays [13, 15, 21, 32], and it has recently been demonstrated also for the stream-bounce display [33]. This shows that perceptual disambiguation also relies on the combination of current sensory information with memory of recent perceptual interpretations [34, 35]. However, it should be noted that this effect might be partially due to the fact that sound and no sound trials were presented in separate blocks, thus stabilizing the percept within each block.

#### Reverse correlation analyses

As already mentioned, the random structure of the dynamic visual noise presented on each trial allows us to isolate the features of the stimulus that modulate the final percept. That is, due to its random fluctuations, noise can sometimes provide signal-like information that might be used by the observer to interpret the ambiguous display. If so, the statistics of the spatiotemporal noise patterns (Fig 1, center) of the stimuli classified as bouncing or streaming should differ. Such properties can be estimated through psychophysical reverse correlation techniques, also known as classification images [28]. To this end, we first calculated the mean (i.e., the average luminance of the noise) and the mean squared error (MSE, the squared-difference from the mean, which is a measure of the contrast energy of the noise) across trials classified one way or the other. We did this calculation for each cell in the space-time noisy stimulus matrix [29]. This procedure was performed separately for those trials classified as bouncing and streaming, to obtain the matrices representing the first (mean) and second order (MSE) statistical properties of the noise selectively associated to either percept (see Methods). The difference between the noise matrices for bounce and stream, known as classification images, represent the templates (or kernels) for perceptual decision making.

The luminance kernel (Fig 2B left; see also S1 Fig for individual participants’ data) indicates how deviations from the mean luminance of each spatiotemporal stimulus sample are associated to the perception of a bounce (as opposed to a stream). The luminance kernel reveals a positive association between luminance along the spatiotemporal trajectory of the moving objects (especially the one moving rightward) and the perception of a bounce. In contrast, the luminance of pixels that do not correspond to the moving target (i.e., the points off the diagonals in Fig 2B, left) should be darker than average for them to trigger more likely a bounce response. In other words, this indicates that light stimuli moving against a dark background are more likely perceived as bouncing (see [36]). Additionally, the luminance kernel has more energy prior to the intersection than after. This demonstrates that visual information before the crossing is especially important in determining the final percept. That is, it does not matter too much for the decision what occurs after the intersection, which makes sense if the perceptual decision is already made at the moment of the intersection.

Next, we analyzed the contrast-related (second order) statistical properties of the stimulus, which are known to modulate performance in both visual and audiovisual tasks [29, 37]. To this end, we calculated the contrast kernel (MSE), representing how the contrast energy of each pixel is associated to a bounce as opposed to a stream response (Fig 2B, right; see S1 Fig for individual participants’ data). The contrast kernel displays a striking similarity with the moving stimulus (Fig 1, left panel), with the contrast of the moving bars (especially the one moving rightward) positively associated to a bounce response. That is, high contrast moving bars are more likely perceived as bouncing. Like in the luminance kernel, the effect of contrast is higher before the intersection.

To phenomenologically demonstrate that these classification images do indeed represent the spatiotemporal stimulus pattern that constitute a stream or a bounce percept, we used the classification images to generate disambiguates stimuli (see S4 Movie and Methods). These displays clearly show that the spatiotemporal patterns of the classification images represent the templates for prototypical streaming or bouncing events, as the stimuli in the video are by-and-large devoid of the intrinsic ambiguity of the standard stream-bounce display (compare S1 Movie and S4 Movie). This was corroborated by showing S4 Movie to a pool of 12 naïve observers, all of which classified the top stimulus as bouncing while the lower one as streaming.

Classification images can also be used to investigate whether sound alters early visual processing. Specifically, if concurrent acoustic stimuli modulate visual processing, the patterns emerging from the classification images for trials with and without sounds should display consistent differences. Therefore, we calculated classification images separately for trials with and without sounds (see S2 Fig, bottom left and middle panels; S3 Fig, bottom left and middle panels). Overall, auditory information had virtually no influence on the shape of the luminance and contrast kernels. This suggests independence between auditory and visual sensory processing [38]–at least as far as it concerns extracting information for perceptual disambiguation.

In a similar fashion, we assessed whether memory of recent perceptual interpretations influenced visual processing, i.e. whether decision on the trial back had any influence on the current decision. To do so, we separately calculated the classification images from trials following a “stream” and a “bounce” response (see S2 and S3 Figs, right panels). Previous responses do not alter the patterns emerging from the classification images for lightness and contrast, hence arguing against an effect of perceptual memory on early sensory processing.

Both classification images display more energy in the rightward direction, and this effect seems rather consistent across participants (see S1 Fig). Perceptual anisotropies are well documented in motion perception [39], and in the present display they may arise either from an asymmetric allocation of visual attention to the moving bars, or leftward-rightward asymmetries in motion processing. As studying perceptual anisotropies is beyond the scope of the current study, we did not further investigate this serendipitous finding.

#### Modeling

A fundamental advantage in using psychophysical reverse correlation analyses with no explicit parametric manipulations of the visual stimuli is that they allow one to generate hypothesis a-posteriori, based on the patterns emerging from the classification images. In this case, reverse correlation analyses reveal that high contrast bars are more likely perceived as bouncing. However, it is not clear why contrast should modulate perception in such a systematic fashion. Studies in visual motion perception demonstrate that, due to the properties of visual motion detectors [40], the perception of motion critically depends on the contrast of the moving objects [41]. This seems to suggest that also visual perceptual disambiguation might rely on the basic filtering properties of motion detection mechanisms.

**Fig 3:**
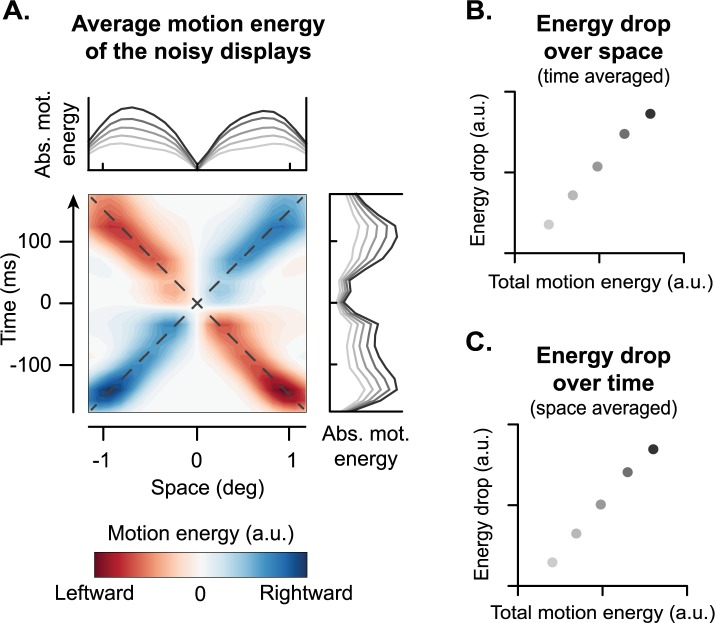
Motion energy analyses. **A**. Average motion energy matrix calculated from the noisy ambiguous displays (note that this is not a classification image). The plots above and to the right of the motion energy matrix represent the motion energy profile averaged over space and time, respectively. Note the drop in motion energy profiles at the intersection of the trajectories. The darkness of the lines represents the amount of total motion energy in the display (darker = more energy). To derive these plots we binned the displays in 5 groups depending on their total motion energy. The drop in motion energy, that is the difference between the maximum and the minimum motion energy of each noisy display, is linearly related in both space (**B**; see also **A**, top plot) and time (**C**; see also **A**, right plot) to the total motion energy–and hence to the contrast–of the display.

To gain insights into the role of motion detection in resolving ambiguity in the stream-bounce display, we used the motion energy model [40], a classic model of visual motion perception (see Methods and S4 Fig). According to this model, humans detect motion and its direction based on a series of spatiotemporally oriented filters, whose output determines the amount of perceived motion and its direction. That is, such model detects what is known as motion energy, a quantity that jointly depends on the speed of motion and the contrast of the moving object with respect to the background. Notably, studies with random-dot kinematograms, demonstrated that the motion energy model can predict human and primate’s performance even when the motion signal was corrupted by external noise (i.e., motion coherence) [42, 43]. Fig 3A shows the average motion energy profile of the noisy moving stimuli (see Methods). Different colors represent the direction of the motion (blue = rightward; red = leftward), and their saturation represents the amount of motion energy. Due to the crossing of the trajectories of the moving stimuli, there is no motion energy at the intersection. To better highlight this, we calculated the motion energy profile over time and space, by integrating the absolute motion energy matrix (i.e., discarding the direction of motion) over space (Fig 3A, right) and time (Fig 3A, top), respectively. The resulting motion profiles show a clear drop in motion energy at the intersection.

Such a drop in motion energy at the intersection might be crucial for perceptual disambiguation: ideally when two non-rigid moving objects collide, their velocity should briefly drop to zero, whereas this should not occur–or at least it should be less evident–in the absence of a collision. Assuming the brain to have knowledge about this generative model, we looked for the footprint of such a perceptual inference in the empirical data. Due to the noise added to the moving stimuli, there should be substantial trial-by-trial variability in the extent of the drop, which could systematically bias perceptual disambiguation. The empirical classification images (Fig 2B) demonstrate that stimuli with high contrast are more likely perceived as bouncing. Therefore, given that contrast modulates motion perception [40], we looked at how changes in motion energy in the noisy stimuli modulated the energy drop at the intersection.

To do so, we calculated the absolute motion energy of each display. This was done by taking the absolute value of motion energy (S4 Fig, bottom) for each display, and averaging the results over both time and space. To better emphasize how the drop in motion energy changes with total motion energy, we binned the stimuli based on their total motion energy and examined how this affected the time-averaged and space-averaged motion energy profile. Results demonstrate that the peak in motion energy increases with increasing total motion energy, while, due to the design of the stimuli, the dip at the crossing remains relatively stable at near-zero motion energy (Fig 3A, top and right plots, darker lines represent more total motion energy). That is, the drop in perceived speed is more pronounced when the total motion energy of the display is higher. Notably, the extent of the drop–calculated as the difference in motion energy between the peak and the dip–is linearly related to the total motion energy of each display (Fig 3B and 3C; see also Fig 3A, note the luminance of the lines in the marginal plots), and hence to the contrast of the moving bars.

This result provides a first hint on the role of motion energy filters in perceptual disambiguation, however this modeling approach further makes a number of predictions that we systematically tested in the current study. First of all, we should be able to predict the percept given the noise pattern in each stimulus on a trial-by-trial basis. Second, if motion energy filters are indeed the underlying mechanisms for visual perceptual disambiguation, the model should also be able to replicate the empirical classification images. Third, given that motion energy filters are not sensitive to the luminance of the moving stimuli, but only to their contrast with respect to the background [40], we should find the same pattern of results even if the moving bars are darker than the background (rather than lighter, like in the current experiment). In the next sections we put the first two predictions to the test, while the third one was tested in a second experiment.

**Fig 4:**
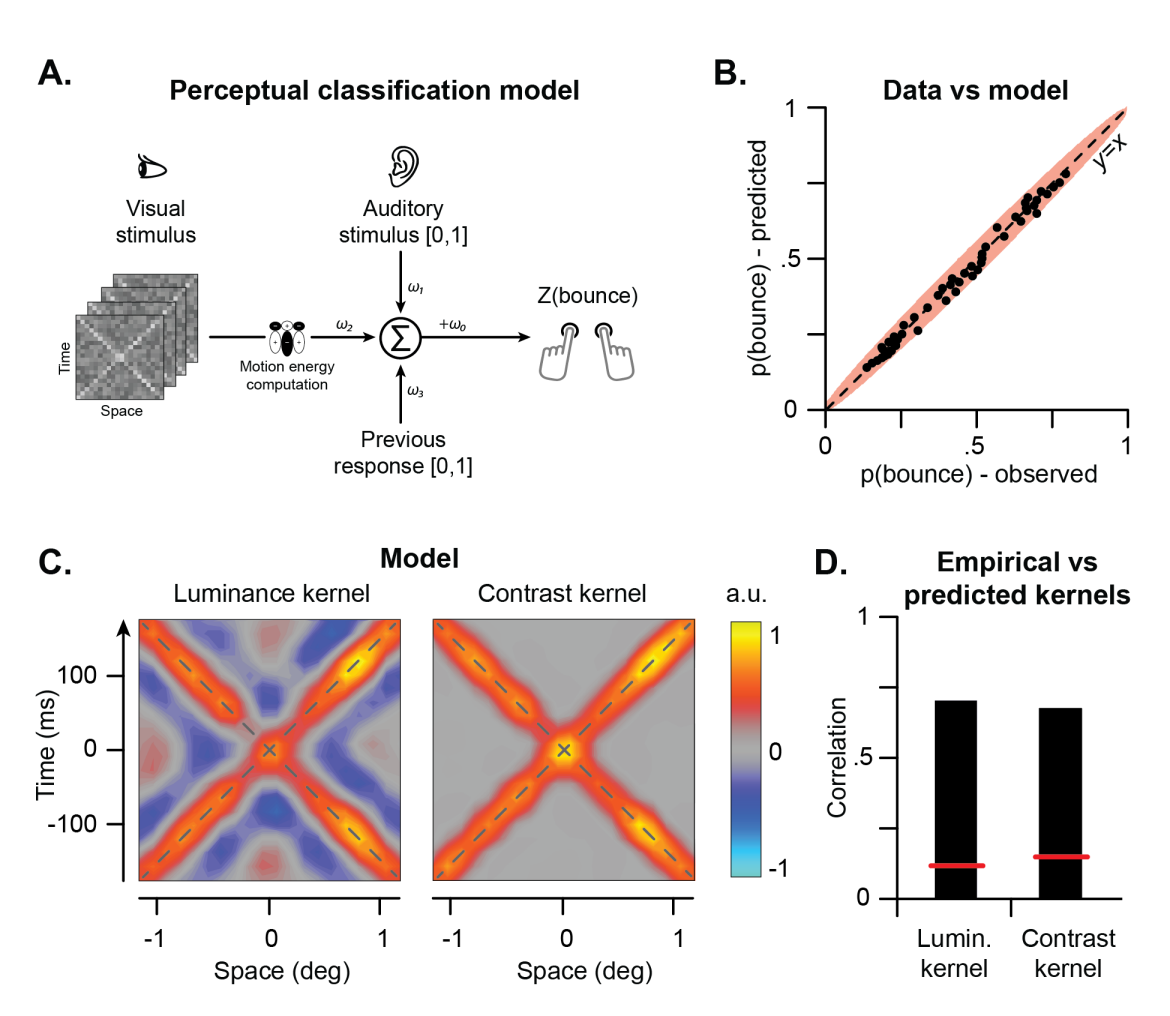
Perceptual classification model. **A**. Model. Visual motion energy is first computed from the stimuli through motion energy filters, and the result is linearly integrated with the auditory information and recent perceptual memory into a single estimate to determine the Z-score of streaming/bouncing responses. **B**. Scatterplot of empirical vs. predicted responses for the aggregate observer. Each dot is the average of 608 responses. Light red area represents the 99% confidence interval of the identity line. **C**. Luminance and contrast kernels calculated from the model responses. **D**. Cross-correlation between predicted and empirical kernels. The red lines represent the thresholds for statistical significance (p = 0.05) as calculated based on the permutation test.

To computationally capture the sensory processes underlying perceptual inference in the stream-bounce display, we developed a simple perceptual classification model (Fig 4A). In the first stage, visual information for perceptual disambiguation is computed for each stimulus in terms of total motion energy. Given that in the present visual display the motion of the bars is identical in both directions, we simply summed the total absolute motion energy (S4 Fig, bottom) to compute, for each noisy stimulus, a measure of the overall motion energy (and hence of the extent of energy drop at the intersection), irrespective of direction (see Methods). The result of such motion energy computation is then combined with auditory information and recent perceptual memory into a single proxy variable representing the overall evidence towards one interpretation or the other. This is done in the following way: In line with current models of sensory integration [36, 44], assuming neural noise to be independent across the channels and normally distributed, we modeled sensory integration of visual motion energy, auditory signals, and recent perceptual memory as weighed linear integration. To make the stream-bounce information provided by the different channels (motion energy, audio signal, memory) directly comparable, linear coefficients *ω*_*i*_ consisted of a combination of both weights and scaling factors [31, 45]. As a consequence, unlike standard weighted averaging models of sensory integration (e.g., [44]), linear coefficients are not constrained to sum to one. This scaled and integrated information represents the evidence towards streaming or bouncing, and eventually determines the final percept. The predictive power of the model was assessed through a cross-validation procedure (see Methods). Overall, the model tightly reproduced the observed responses (Fig 4B, see S1 Fig, right column, for single observers’ data).

To further validate the current model, and to test whether motion energy is indeed the primary visual cue underlying perceptual disambiguation in the stream-bounce display, we calculated the classification images for both luminance and contrast based on the responses of the model. This was done using the classification responses provided by the model (see before). The results (Fig 4C) show a remarkable similarity with the empirical classification images (Fig 2B) and this was true for both the luminance and the contrast kernels. Notably, the model could even replicate the fine details of the empirical classification images, including the regular alternation of positive and negative peaks that we found in the original data (compare Fig 2B and Fig 4C). The main difference between predicted and empirical kernels is that the empirical classification images are rather asymmetric, and assign more weight before the crossing. To formally quantify such a similarity, we computed the normalized pixel-by-pixel correlation between the empirical and the predicted classification images for both luminance (ρ = 0.71) and contrast (ρ = 0.69, Fig 4D). These correlations were highly significant, as assessed using a permutation test whereby we randomly permuted the value of each sample of the classification images over 20,000 iterations. Notably, a simpler model that only responds to contrast (but not to motion), was not sufficient to replicate the current findings (see Methods).

It is important to note that this model also reveals why the manipulation of the timing and luminance of the moving stimuli in the study by Zhou and colleagues [21] modulated perceptual disambiguation in such a systematic fashion. This is because both manipulations varied the motion energy drop of the stimuli at the intersection.

### Experiment 2

**Fig 5:**
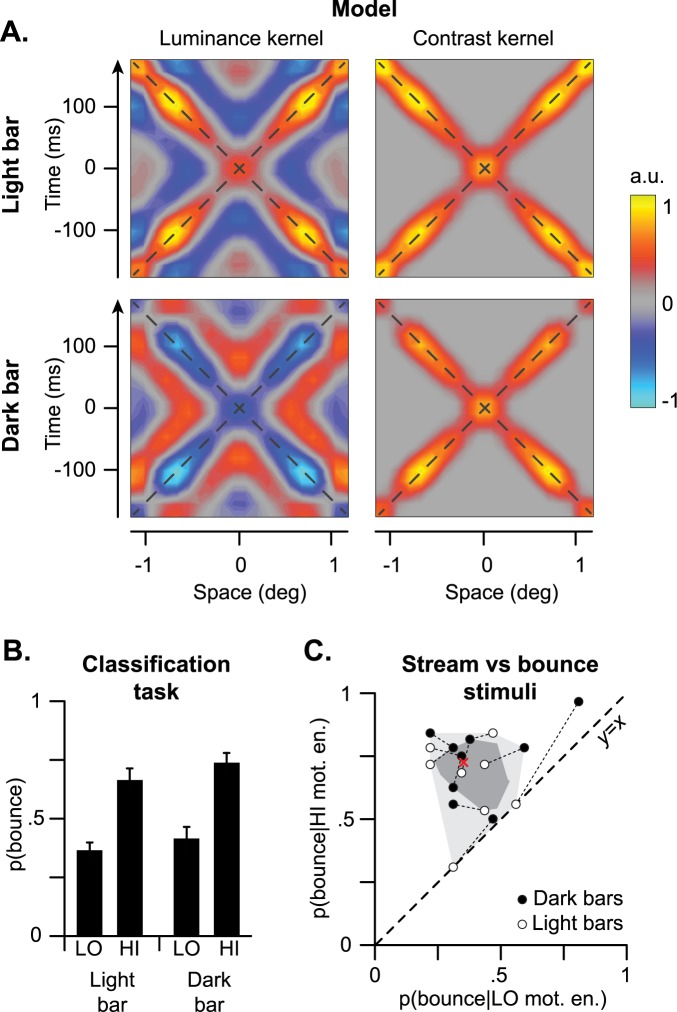
Experiment 2. **A**. Luminance and contrast kernels estimated from the motion energy model for light (top) and dark (bottom) moving bars. **B**. Results of Experiment 2. The bars represent the probability of responding bounce for high (HI) and low (LO) motion energy drop and for light and dark bars. Errorbars represent the standard error of the mean. **C**. Scatterplot and bagplot of the probability of responding bounce for stimuli with high and low motion energy drop. Thin dashed lines connect data from the same participants in the two lightness conditions. The red cross represents the depth median.

The responses of motion energy filters are strongly modulated by the contrast of the moving object, while being relatively insensitive to luminance. Therefore, a critical test for the role of motion energy filters in disambiguating the stream-bounce display would be to invert the luminance polarity of the moving bars with respect to the background, while keeping their contrast constant. If motion energy computation is indeed the underlying mechanism for perceptual disambiguation, it should not matter whether the moving bars are lighter than the background (like in the previous experiment) or darker. Rather, what would matter should be the amount of the drop of motion energy at the intersection, which correlates with the total motion energy in the display. To directly test this hypothesis, we generated stimuli analogous to the ones used in the previous experiment, but with an additional modulation of both the total motion energy and the luminance polarity of the moving bars (Fig 5A and S3 Movie, see Methods for further details). Next, we run a psychophysical task where we asked participants to classify such displays as streaming or bouncing, with the hypothesis that stimuli with high motion energy, and hence with a large drop of motion energy at the intersection, should be more likely classified as bouncing, irrespective of the luminance polarity of the moving bars.

As hypothesized, displays with a large drop in motion energy (i.e., high motion energy) were systematically classified as bouncing more often than those with a smaller drop (i.e., low motion energy, Fig 5B), whereas luminance did not significantly affect participants’ responses. Notably, the magnitude of the effect of motion energy drop on perceptual disambiguation was comparable to the effect of sound presence/absence in Experiment 1 (compare Fig 2C left, and Fig 5B), and it was highly consistent across participants (Fig 5C). This result demonstrates that motion energy, and its drop at the intersection, is indeed the key visual factor driving perceptual disambiguation, and further validates the current classification model.

## Discussion

The mechanisms underlying perceptual disambiguation are a central topic in sensory neuroscience. Resolving ambiguity requires both extracting and combining sensory information. The present results demonstrate which cues are relevant to resolve perceptual ambiguity in the stream-bounce display, and highlight the mechanisms underlying both the computation and the combination of such multisensory cues for the perception of dynamic ambiguous displays. Previous research has already investigated the stimulus properties biasing the interpretation of the stream-bounce display (e.g., [36, 38, 46]). Such studies relied on a parametric manipulation of one or more stimulus features. However, this approach requires researchers to decide a priori which features might be relevant to solve the task. The key advantage of using noisy stimuli and reverse correlation analyses relies on the absence of any prior assumptions, which allows us to determine a-posteriori how random fluctuations in the (external) noise systematically modulate participants’ responses. This, in turn, allows gathering detailed information about the precise spatiotemporal features buried in the stimuli that underlie perceptual disambiguation. Such key features and their relative contribution to perceptual decision making can only be obtained using standard psychophysical procedures by lucky guessing, and it is never clear whether some key features have been missed. An example from this study is the importance for disambiguation of motion energy, and its drop at the intersection, which might not have been discovered with traditional psychophysical methods. What is more, reverse correlation analyses revealed that sound does not alter early visual processing; rather, it modulates the percept after the unimodal information for disambiguation has been independently extracted from all modalities.

Over the last decade, multisensory integration has often been modeled in terms Bayesian decision theory. Empirical results demonstrate that the brain operates in a statistically optimal way by maximizing the accuracy and precision of combined sensory estimates when integrating redundant and unambiguous sensory information [30]. Before integrating multisensory information for perceptual disambiguation, however, the brain needs to transform sensory information into a common format to make it directly comparable. That is, the different signals should be separately processed to extract stream-bounce information (i.e., probability of impact) from the continuous stream of sensory signals. Here, we modeled this transformation in terms of motion energy filters [40], which transforms complex, dynamic visual information into proxy decision variables that represent the strength of sensory evidence. Once transformed into a common format, sensory evidence from vision and audition can be directly compared and integrated by weighted averaging. A simple linear integration model (without interactions across the cues) captures human perception with a high degree of accuracy [36]. This result demonstrates that the brain applies analogous integration principles for disambiguation as it does for integrating redundant information [44]. What is more, the close correspondence between the empirical classification images and those predicted based on the motion energy model, further supports the motion energy model itself.

A pressing question in the study of perceptual ambiguities concerns the conditions or parameters that drive perceptual biases. That is, why on each trial a given interpretation is chosen over the competing one. Internal noise has often been advocated to explain perceptual alternation [19, 21–24]. Namely, noise was advocated as causing perceptual switches during prolonged presentations of bistable stimuli, like in binocular rivalry. However, to date we still do not know exactly which spatiotemporal (i.e., “signal-like”) patterns within the noise selectively drive perceptual disambiguation between two equally valid alternatives. By embedding the stimulus in external noise with known properties, and using reverse correlation analyses, this study demonstrates not only that noise is indeed the key element driving alternation, but also which pixel-by-pixel properties of the noise are systematically associated to each interpretation. More specifically, due to its random structure, noise often contains information that is used in the process of resolving ambiguity. Here, we characterize what such properties are in the case of the stream-bounce display, and how they get extracted though spatiotemporal visual filters. Notably, the existence of systematic links between low-level stimulus properties and perceptual responses–as measured through reverse correlation analyses–argues against interpretations of disambiguation purely in terms of attention or response biases [47–50].

In recent years, the stream-bounce display has often been used to investigate the neural underpinnings of multisensory integration. The main findings support the involvement of multimodal cortical regions [51] and large-scale synchronizations of oscillatory neural activity [52, 53] in resolving multisensory perceptual ambiguity. However, the computational principles underlying the multi-stability of the stream-bounce display remained poorly understood. The current results fill this important gap and provide evidence for the fundamental role of motion energy computation and linear integration of evidence in multisensory perceptual disambiguation.

## Methods

### Psychophysical experiment

Three participants (2 naïve females, VL and CG, and one male, the author CP) took part in Experiment 1. All participants were right handed and had normal or corrected to normal vision and audition. Participants sat in front of a computer screen with their head constrained by a chin and headrest. Each trial started with the presentation of a red fixation cross at the center of the screen (600ms), after which the visual stimulus appeared, consisting of two light vertical bars (0.085° x 0.426° each) moving in opposite direction and embedded in dynamic visual noise. The dynamic stimulus comprised 20 frames (60Hz screen, overall duration 333ms) of uni-dimensional visual noise consisting of 20 vertical bars (0.085° x 0.426° each) with random luminance. The luminance of each noise sample varied between 14.6 cd/m^2^ and 48.3 cd/m^2^. The moving visual stimuli were defined by a 50 cd/m^2^ luminance increment. The stimuli used in Experiment 1 are contained in S1 Dataset. On half of the blocks, a 16ms white noise auditory click was played from two speakers flanking the screen when the two moving stimuli met at the center of the screen. Participants were informed about the presence or absence of the sound at the beginning of each block. Such a blocked design was necessary as in preliminary observations we found that when sound and no-sound trials alternated randomly, participants’ responses were almost-exclusively determined by the presence/absence of the sound. Therefore, a blocked design made it easier to empirically estimate visual classification images.

The relatively small size and short duration of the stimuli were selected to discourage eye-movements. Observers’ task was to look at the stimuli without moving their eyes, and to report by a key-press whether they perceived the stimuli as bouncing or streaming through each other. Participants were explicitly told that there was no correct or wrong answer. The experiment was performed in a dark anechoic chamber and it was controlled by a custom-built software based on the Psychtoolbox 3 [54]. Experiment 1 consisted of ~10,000 trials per participant (CP: 10240 trials; CG: 10320; VL: 9840 trials). Psychophysical data is contained in S2 Dataset. Sound significantly modulated the percept (overall: χ^2^ = 3404; p<0.001; CP: χ^2^ = 584; p<0.001; CG: χ^2^ = 1503; p<0.001; VL: χ^2^ = 1491; p<0.001): only 27% (CP: 36%; CG: 21%; VL: 23%) of the trials without sound were perceived as bouncing, against 61% (CP: 60%; CG: 59%; VL: 62%) of the trials with sounds (Fig 2C). Also, participants had a strong bias towards interpreting the stimulus just as they did in the previous trial (overall χ^2^ = 2693; p<0.001; CP: χ^2^ = 531; p<0.001; CG: χ^2^ = 1818; p<0.001; VL: χ^2^ = 547; p<0.001). Given that we were interested on the effects of the previous response, the first trial of each block (of 40 trials) was discarded from these analyses.

In Experiment 2 we used the motion energy model to generate noisy stream-bounce displays–with dark and light moving bars–with a parametric manipulation of motion energy (and hence of motion energy drop, see S3 Movie). This was achieved by first calculating classification images based on total motion energy for noisy visual stimuli with both light and dark moving bars. To estimate the classification images for motion energy, we generated a series of 400,000 noisy stimuli like in the previous experiment, with the only difference that the moving bars could be either lighter or darker than the background. Such stimuli were then fed into the motion energy model (see below) to calculate their total motion energy (i.e., the sum of rightward and leftward motion energy), and finally we discretized the models’ response by classifying the 50% of the stimuli with higher motion energy as bounce and the remaining ones as streaming. This procedure was separately performed for stimuli with light and dark bars. Classification images were then calculated following the same procedure used in Experiment 1. The resulting classification images looked similar to those of Experiment 1 (Fig 4A), however, the luminance kernels for light and dark bars had opposite polarities.

Such classification images were then used to create stream-bounce displays with high or low motion energy (S3 Movie). First off, we generated a series of stimuli similar to the ones used in Experiment 1, but again the moving bars could be either lighter or darker than the background. Then, we experimentally manipulated the amount of motion energy by either adding or subtracting the luminance kernels to obtain displays with high or low motion energy, respectively. This procedure was separately performed for light and dark bars using their respective classification images. Such manipulated displays were then used in a psychophysical classification task in Experiment 2.

Overall, the task was very similar to Experiment 1. However, in Experiment 2 we did not play any sounds. Visual stimuli with high and low motion energy randomly alternated across trials, while light and dark bars were presented in separate blocks of 16 trials each. In total, the experiment consisted of 126 trials. That is, 32 trials for each of the four combinations of bars’ luminance (light vs. dark) and total motion energy (high vs. low). Ten naïve participants (7 females) took part in Experiment 2. Compared to Experiment 1, the larger number of participants in Experiment 2 was due to the fact that in the latter experiment each participant performed a much smaller number of trials (126 vs. ~10,000 trials). Before starting the experiment, all participants underwent a short practice session to familiarize with the stimuli and the task.

The probability of reporting a bounce for each condition and participant was normalized through a Z-score transformation and was analyzed using a 2x2 repeated-measures ANOVA with motion energy and luminance as within-participants factors. Motion energy strongly modulated participants’ responses (F(1,9) = 35.489, p<0.001), with no effects of luminance (F(1,9) = 2.317, p = 0.162) and no interactions (F(1,9) = 1.036, p = 0.335).

This study was conducted in accordance with the Declaration of Helsinki and the experiments had ethical approval from the ethics committee of the University of Tübingen.

### Reverse correlation analyses

To calculate visual classification images we first sorted the noisy visual stimuli presented in the experiment according to participants’ (or model’s) classification responses (stream vs. bounce). For each class, we calculated the mean luminance (*μ*) and contrast (mean squared error, *MSE*) of the noisy visual stimuli and we combined them as follows to obtain the classification images for visual luminance (*K*_*L*_) and contrast (*K*_*C*_):

$$K_{L}(x,t)=\mu_{\left[bounce\right]}(x,t)-\mu_{\left[stream\right]}(x,t)$$ (1)

$$K_{C}(x,t)={MSE}_{\left[bounce\right]}(x,t)-{MSE}_{\left[stream\right]}(x,t)$$ (2)

Where *μ*_[*R*]_ and *MSE*_[*R*]_ are the mean and the mean squared error templates for the stimuli *S*_[*R*]_, respectively. *R* denotes participants’ responses. Visual classification images (*K*_*L*_, *K*_*C*_) were smoothed by convolution with a low-pass spatiotemporal filter of the form [0.49, 0.7, 0.49; 0.70, 1.0, 0.70; 0.49, 0.7, 0.49] [55]. Finally, all classification images were range-scaled so that their maximum absolute values equal to 1.

Classification images were calculated individually for each participant and on the aggregate observer obtained by combining data from all participants.

### Creating unambiguous stream-bounce displays

As a proof-of-principle to demonstrate that the classification images really represent the templates of prototypical streaming or bouncing events, we reverse engineered the stimuli, and used the classification images to create unambiguous stimuli. To do so, we first generated noisy stimuli like in Experiment 1. Next, we added or subtracted the empirical luminance kernel of the aggregate observer of Experiment 1 (Fig 2B) to modulate luminance over time and space, and hence to generate disambiguated ‘bouncing’ and ‘streaming’ stimuli, respectively. The resulting stimuli (S4 Movie) provide a striking example of unambiguous dynamic displays: the “bouncing” stimulus (S4 Movie, top) is most likely perceived as bouncing, while the “streaming” stimulus (S4 Movie, bottom) is mostly seen as streaming. This was corroborated by showing the video to a pool of 12 naïve participants (3 female) and asking them which of the two stimuli appeared to be streaming and which bouncing. As expected, all participants indicated the top stimulus as bouncing and the lower one as streaming. This further corroborates the validity of the present reverse correlation analyses, and phenomenologically demonstrates that the empirical classification images do indeed represent the templates for prototypical streaming or bouncing events.

### Modeling

The extraction of visual sensory evidence *E*_*v*_ for perceptual classification is modeled in terms of total motion energy (see below). In order to keep the model simple and because characterizing early stages of sensory processing is beyond the scope of the current study, we assumed the stimuli to be linearly transduced.

The integration of task-relevant information *E* was modeled in terms of weighted linear summation of the form:

$$Z(bounce)=\omega_{0}+\underset{i}{\sum }\omega_{i}E_{i},$$ (3)

where *ω*_*i*_ denotes the linear coefficient (i.e., the weight), *E*_*i*_ the evidence, *ω*_0_ the bias (i.e., the decision criterion), and the subscripts *i* the source of the evidence (i.e., visual motion energy, auditory click, previous response). An assumption of this model is that the internal noise for each task-relevant evidence *E*_*i*_ is independent and normally distributed.

Such a linear model has one covariate *E*_*v*_ corresponding to the evidence from visual motion energy and two factors *E*_*A*_ and *E*_*R*(*t*−1)_ denoting the presence or absence of a sound and the response given on the previous trial. The coefficients of the model were fitted individually for each participant and for the aggregate observer using the Matlab routine *glmfit*. Given that we were interested in understanding the effect of the previous trials on subsequent responses, the first trial of each block was not used for modeling purposes.

The model was validated using a 39-fold cross-validation procedure (for both individual and aggregate observers). The training set was used on each iteration to fit the coefficients of the model. Next, we fed the stimuli of the test set into the model and we compared the response of the model to the empirical data. Each trial was included in the test set in only one iteration. To evaluate how well the model could reproduce the observed responses, we partitioned all trials in 50 bins according to the model response *Z*(*bounce*) in the test set of the cross-validation. The predicted probability of reporting a bounce on each trial, *p*(*bounce*), was calculated from the models’ response using the following equation:

p(bounce)=Φ[Z(bounce)] (4)

where Φ[∙] is the cumulative normal distribution function. For each bin, we also calculated the observed probability of reporting a bounce and we plotted predicted vs. observed responses (Fig 4B, S1 Fig right column). If the model accurately captures participants’ responses, data should lie along the identity line. The 99% confidence interval along the identity line was calculated based on the binomial distribution and the number of responses of each bin. Overall, the model could replicate observed responses with high accuracy.

The current linear integration model was compared to alternative models generated by taking only a subset of the three predictors (i.e., motion energy, sound, or previous response) or by also including interaction terms. The selected model outperformed all such alternatives, as assessed in terms of Akaike information criterion.

### Motion energy model

The motion energy model [40] is a classic and biologically plausible model of visual motion detection based on the combination of a series of spatiotemporally tuned filters. Although a full description and rationale of the motion energy model can be found elsewhere (e.g., [40, 56], here we briefly describe its main features and provide the equations of the spatial and temporal filters. In the current study we implemented a recent version (including the values of the relevant parameters) of the motion energy model [56]. Motion filters had the same spatial and temporal extent as the visual stimuli and they were sampled at the same spatial and temporal resolution. The spatial filters consisted of even (E) and odd (O) Gabor functions:

$$E(x)=cos(2\pi fx)∙e^{{-(\frac{x}{\sigma })}^{2}}$$ (5)

$$O(x)=sin(2\pi fx)∙e^{{-(\frac{x}{\sigma })}^{2}}.$$ (6)

The spatial constant *σ* was 0.5 deg and its spatial frequency *f* was 1.1 cpd. Such values approximate the spatial sensitivity of the magnocellular system [57].

Temporal filters were defined by the following equation:

$$R(t)={\left(kt\right)}^{n}∙exp(-kt)∙[1∕n!-\beta {\left(kt\right)}^{2}∕(n+2)!]$$ (7)

The parameter *k* represents the center temporal frequency of the filter and its value was set to 100. The parameter *n* represents the temporal constant of the filter and its value was set to 9 for the slow temporal filter and 6 for the fast one. The parameter *β* represents the weighting of the negative relative to the positive phase of the filter and its value was set to 0.9.

The model also includes a normalization step. A graphical representation of the full model is displayed in S4 Fig and a Matlab implementation of the model is available online (http://www.georgemather.com/Code/AdelsonBergen.m).

In the current study, the total motion energy of each stimulus was calculated. The motion energy matrix displayed in Fig 3 was obtained by computing the motion energy matrices from all the stimuli presented in the experiment, and averaging the results.

### Alternative model

Given that the results of Experiment 1 point to the role of contrast in perceptual disambiguation, we assessed whether a simpler model which is sensitive to contrast but not to motion is sufficient to explain the current results. For this, we implemented a model consisting of two biphasic spatial filters in quadrature pair with a Gaussian temporal profile. Such a model has been adopted, and fully described, in a related study which also relied on reverse correlation analyses [29]. The spatial filters (and the values of the relevant parameters) of this model are identical to those of the energy model presented here, and based on [29] we set standard deviation of the Gaussian temporal filter to 40ms. We used this alternative model to calculate the predicted classification image, just as we did for the motion energy model. S5 Fig shows that this simpler model, which does not respond to motion, simply cannot account for human performance.

## Supporting information

S1 Fig Single observer analyses. Results of Experiment 1 for each individual observer (from top CP, CG, VL) and for the aggregate observer (bottom). See Fig 2 for further details.
(EPS)

S2 Fig Effect of noise and previous response on the luminance kernel. Classification images for luminance calculated separately for sound presence/absence and for trials following a stream/bounce response (aggregate observer) in Experiment 1. The bottom-right panel corresponds to the classification image presented in Fig 2.
(EPS)

S3 Fig Effect of noise and previous response on the contrast kernel. Classification images for contrast calculated separately for sound presence/absence and for trials following a stream/bounce response (aggregate observer) in Experiment 1. The bottom-right panel corresponds to the classification image presented in Fig 2.
(EPS)

S4 Fig The motion energy model. The stimulus matrix is convolved by a series of spatiotemporally tuned filters, whose output are combined, squared and normalized to calculate leftward and rightward motion energy. Rightward and leftward energy matrices are subtracted to compute the opponent energy matrix (see Fig 3).
(EPS)

S5 Fig Alternative model. Luminance and contrast kernels calculated from the alternative model. This model is sensitive to contrast but not to motion, and it is unable to replicate the empirical classification images (see Fig 2B).
(TIF)

S1 Movie Stream-bounce display. Switch the audio volume on and off to assess the effect of sound on perception; we recommend using VLC player.
(AVI)

S2 Movie Stimuli used in Experiment 1. A complete description of how the stimuli were generated can be found in Fig 1. Switch the audio volume on and off to assess the effect of sound on perception; we recommend using VLC player.
(AVI)

S3 Movie Stimuli used in Experiment 2. The upper display has more motion energy than the lower display. Dark and light moving bars alternate in the movie. To better appreciate the effect of motion energy on perception, it is recommended to look at one display at a time (either the upper or lower one), while covering the other: the upper display should appear to bounce more often than the lower display. We recommend using VLC player.
(AVI)

S4 Movie Disambiguated stimuli. To better appreciate the effect, it is recommended to look at one display at a time (either the upper or lower one), while covering the other: the upper display should appear to bounce more often than the lower display. We recommend using VLC player.
(AVI)

S1 Dataset Stimuli. Each layer of this 3D matrix contains the time-space luminance diagram of the stimuli presented in each trials (e.g., see Fig 1 bottom-right). Each row represents one video frame (first row is the first frame). Columns represent the spatial location of each element in the display (first column is left). The different layers represent different trials, and they correspond to the rows of the data matrix. Each cell in the 3D matrix represents the luminance of each element in the display (cd/m^2^). Data is available as a Matlab MAT-file.
(MAT)

S2 Dataset Data matrix. The first column contains the ID of the observer (CP = 1; CG = 2; VL = 3). The second column indicates the presence/absence of the sound (1 = present). The third column contains the response of the previous trial (1 = bounce). The fourth column indicates the order of the trial within each block (given that we were interested in the effects of the previous response, the first trial of each block is not included). The fifth column contains the response (1 = bounce). The sixth trial contains the reaction time (in seconds). Data is available as a Matlab MAT-file.
(MAT)
