# Supplementary figures and images for: Noise, multisensory integration, and previous response in perceptual disambiguation
Source: PLoS Comput Biol. 2017 Jul 10;13(7):e1005546. doi: 10.1371/journal.pcbi.1005546 (PMC5524419; doi:10.1371/journal.pcbi.1005546)

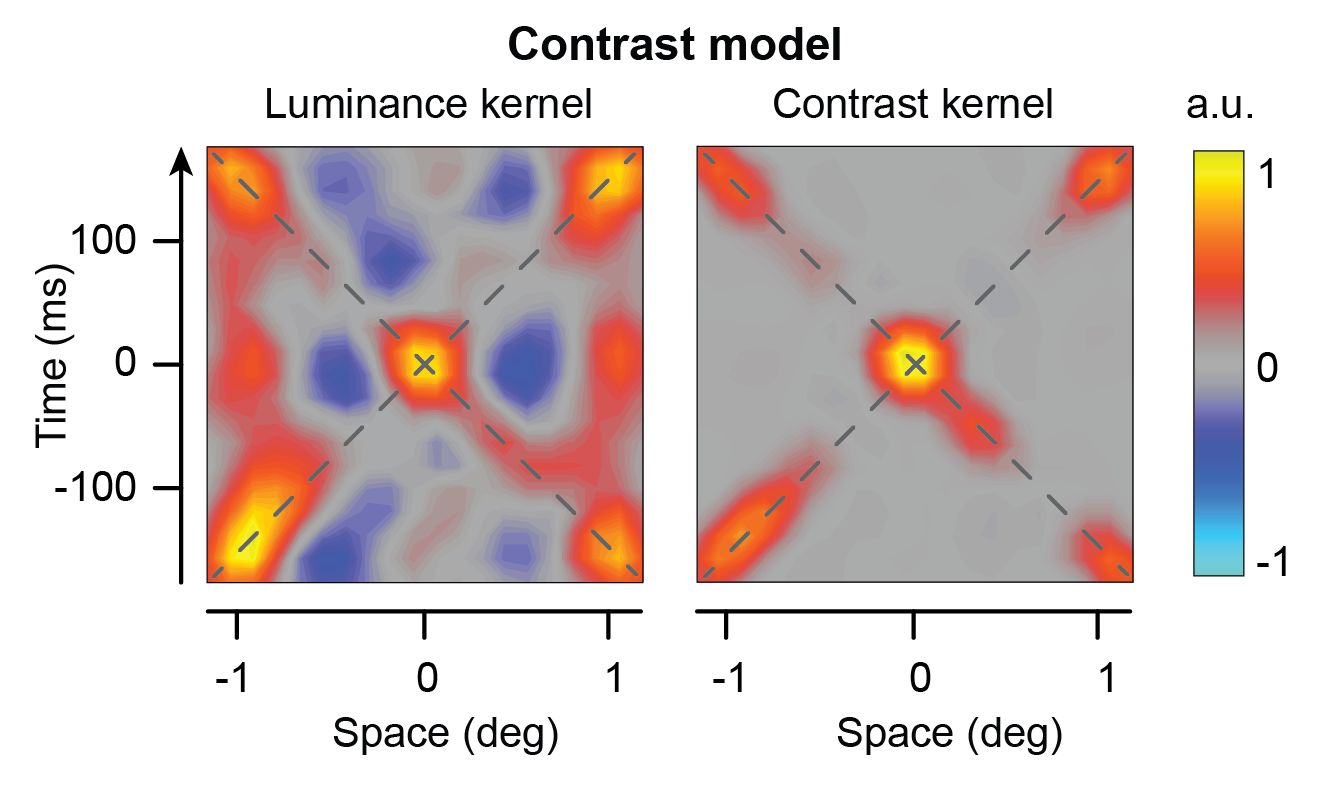

Supplement: S5 Fig — Luminance and contrast kernels calculated from the alternative model. This model is sensitive to contrast but not to motion, and it is unable to replicate the empirical classification images (see Fig 2B). (TIF) [file pcbi.1005546.s005.tif]
